# Supplementary material for: Fullerenols Prevent Neuron Death and Reduce Oxidative Stress in Drosophila Huntington’s Disease Model
Source: Cells. 2022 Dec 31;12(1):170. doi: 10.3390/cells12010170 (PMC9818496; doi:10.3390/cells12010170)
Supplement: Supplementary file 1 [file cells-12-00170-s001.zip › cells-2100617-supplementary.pdf]

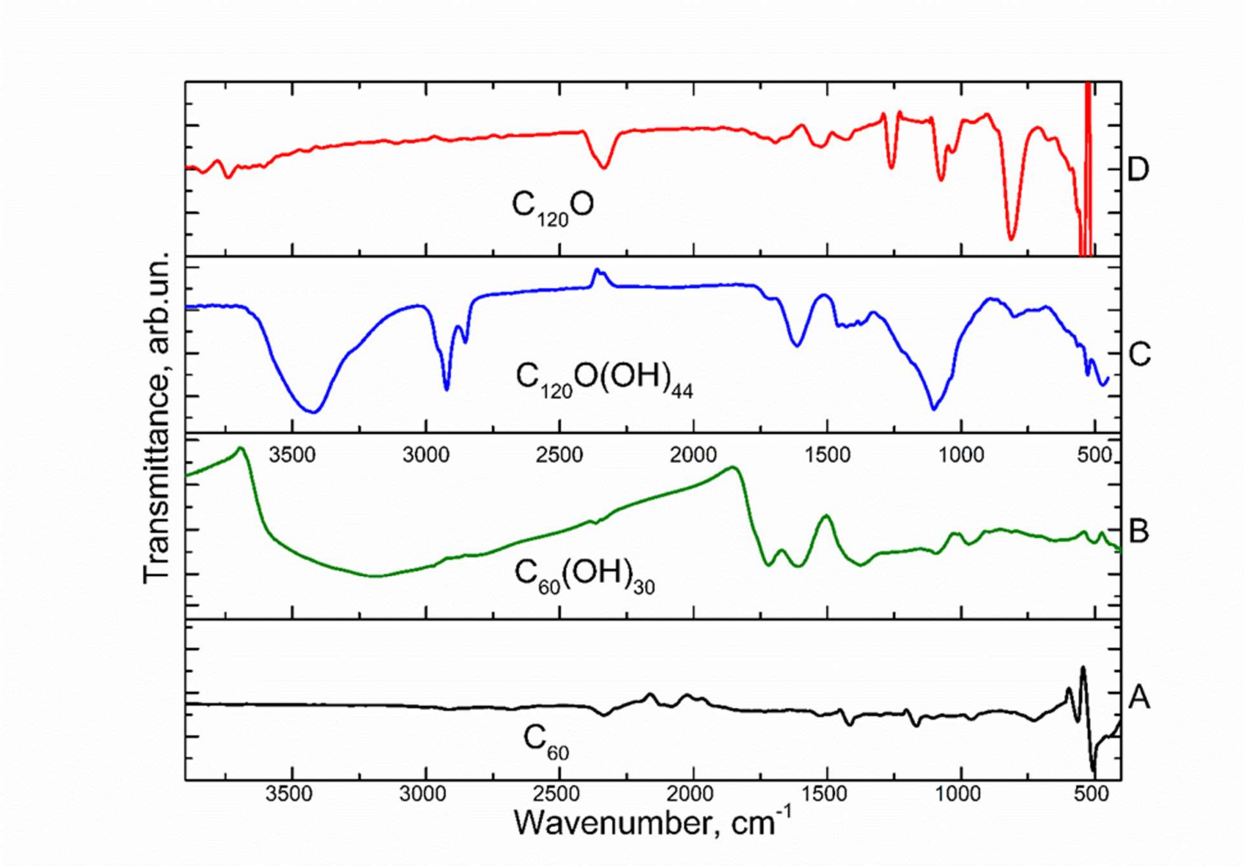

Figure S1. FTIR spectra of pristine C_{60} (A), fullerenol $C_{60}(OH)_{30}$ (B), fullerenol $C_{120}O(OH)_{44}$ (C) and $C_{120}O$ (D)

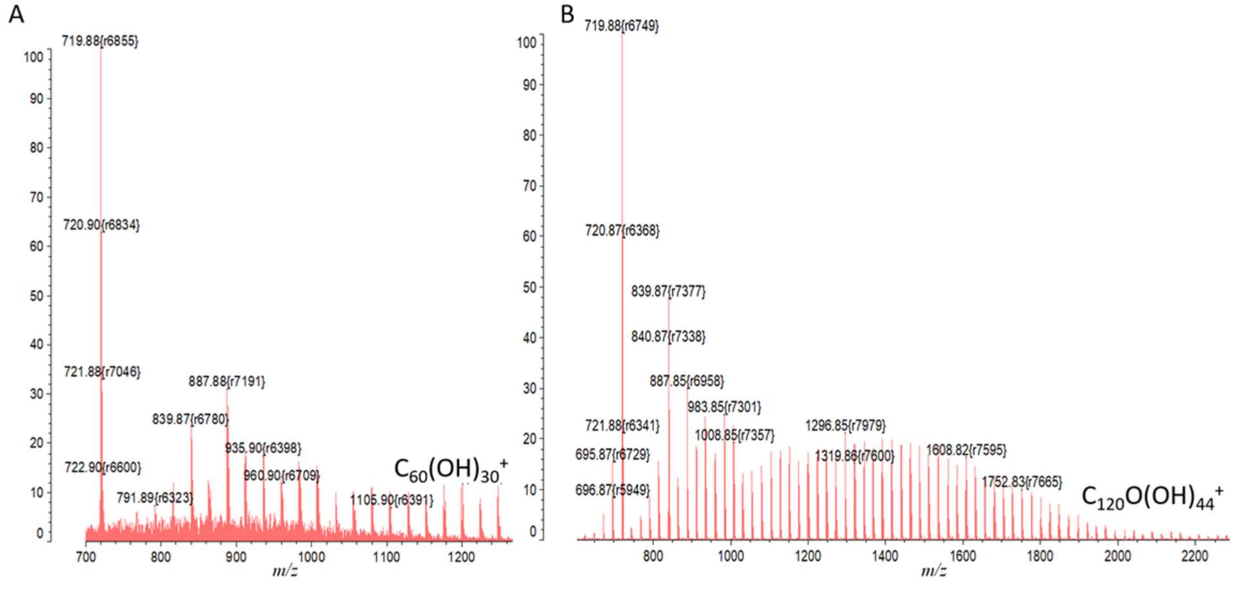

Figure S2. MALDI-TOF/TOF spectrum of fullerenol $C_{60}(OH)_{30}$ (A) and $C_{120}O(OH)_{44}$ (B).

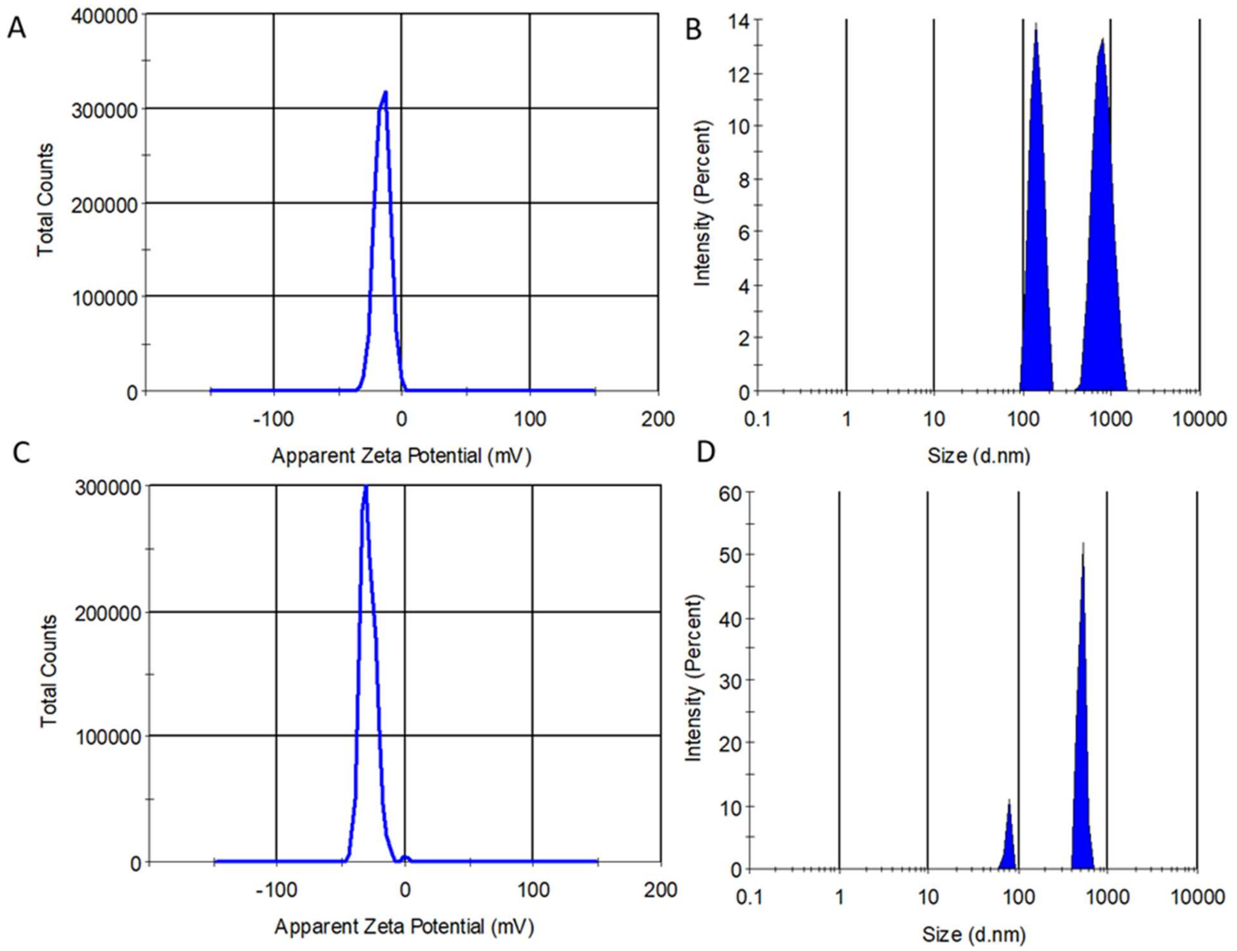

Figure S3**.** Zeta potential and **s**ize distribution of fullerenols particles from $C_{60}(OH)_{30}$ (A, B) and $C_{120}O(OH)_{44}$ (C, D)

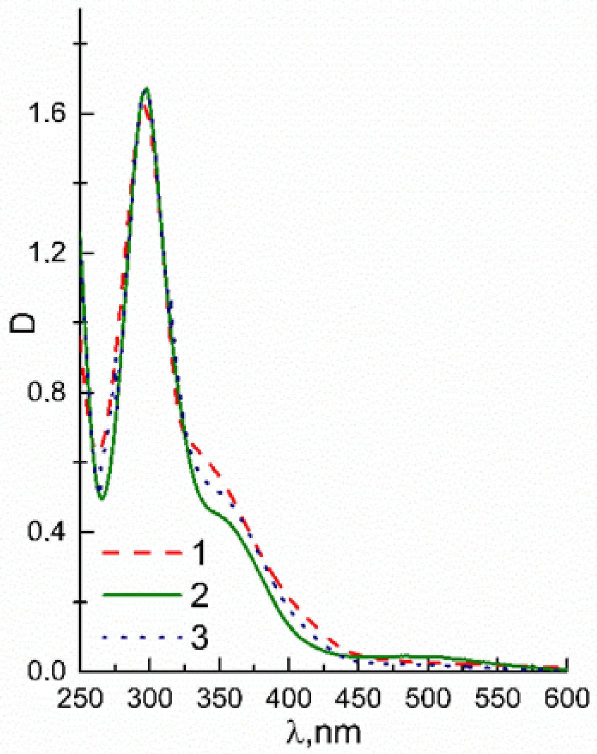

Figure S4. The optical absorption spectra of adrenaline autoxidation products at 5 min of the reaction: 1 – pure adrenaline (control); 2 - adrenaline and fullerenol $C_{60}(OH)_{30}$; 3 – adrenaline and fullerenol $C_{120}O(OH)_{44}$

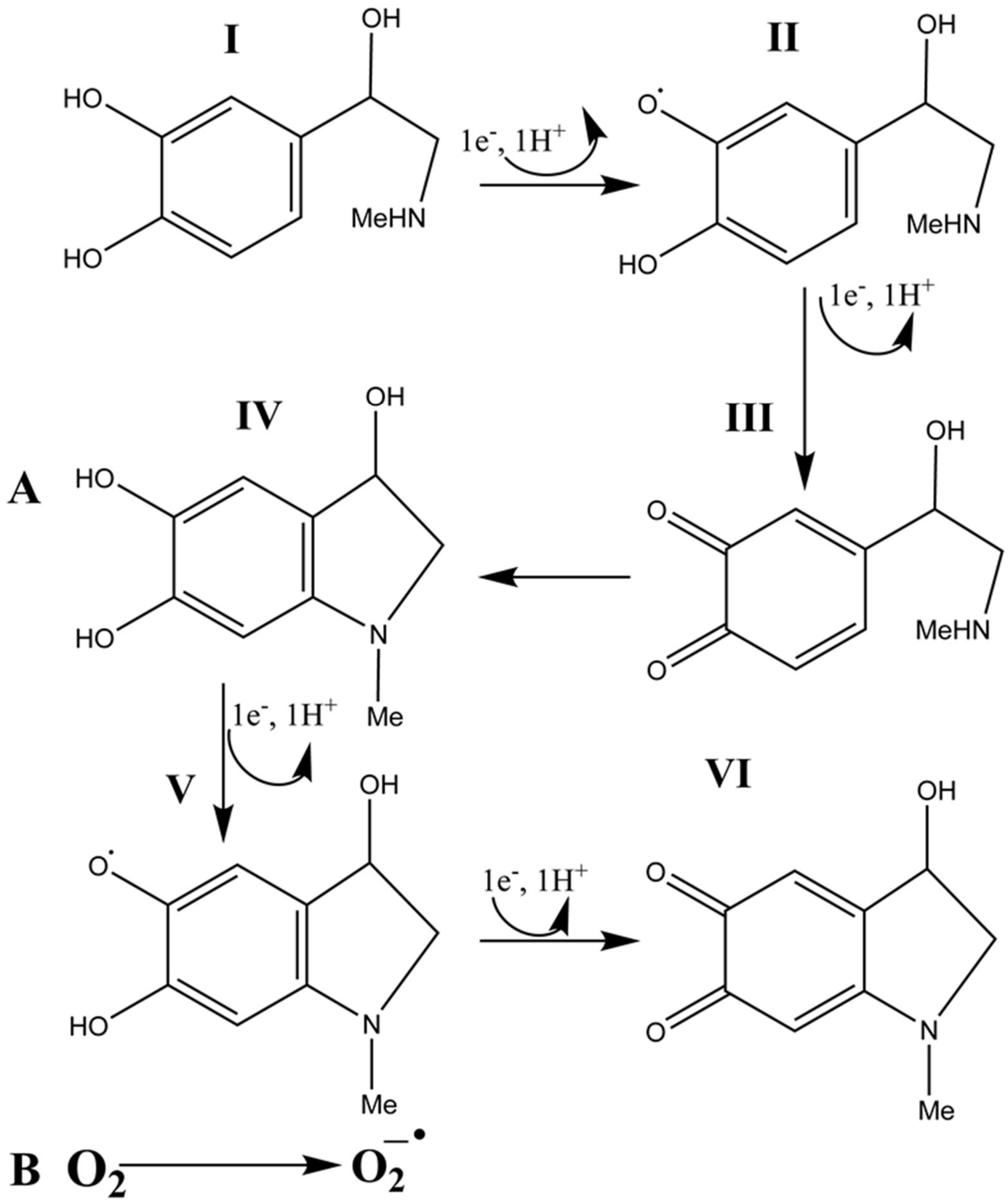

Figure S5. Scheme of the formation of adrenochrome (A) and superoxide anions (B) in the process of autoxidation of adrenaline along the quinoid pathway. I - adrenaline; II - adrenaline semiquinone; III - adrenaline quinone; IV - leukoadrenochrome; V, adrenochrome semiquinone; VI – adrenochrome.

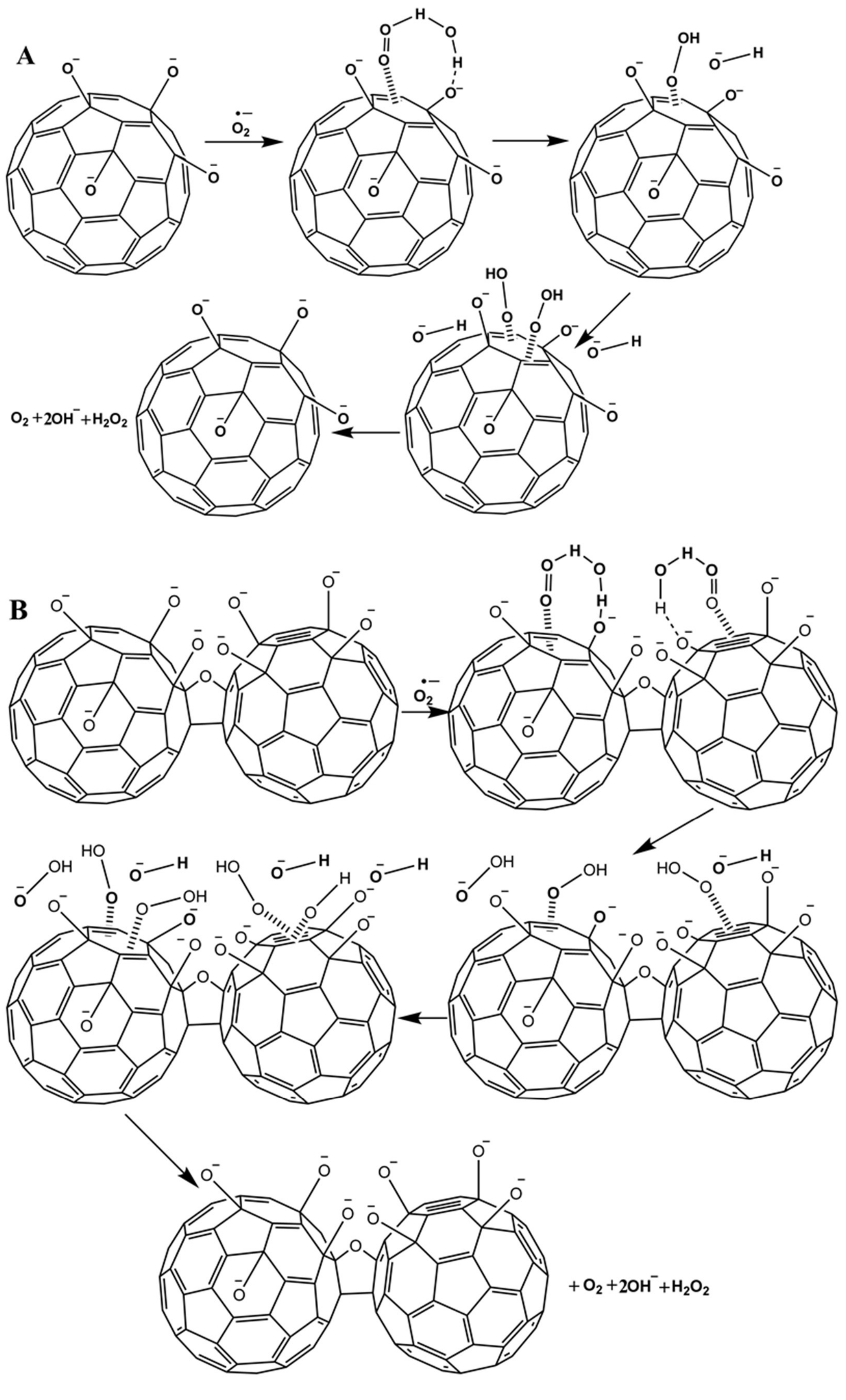

Figure S6. The possible $O_2^{\bullet-}$-scavenging mechanism for fullerenols $C_{60}(OH)_{30}$ (A) and fullerenol $C_{120}O(OH)_{44}$ (B). Adapted from [Wang, Z.; Wang, S.; Lu, Z.; Gao, X. Syntheses, Structures and Antioxidant Activities of Fullerenols: Knowledge Learned at the Atomistic Level. *J Clust Sci* **2015**, *26*, 375–388, doi:10.1007/s10876-015-0855-0.].

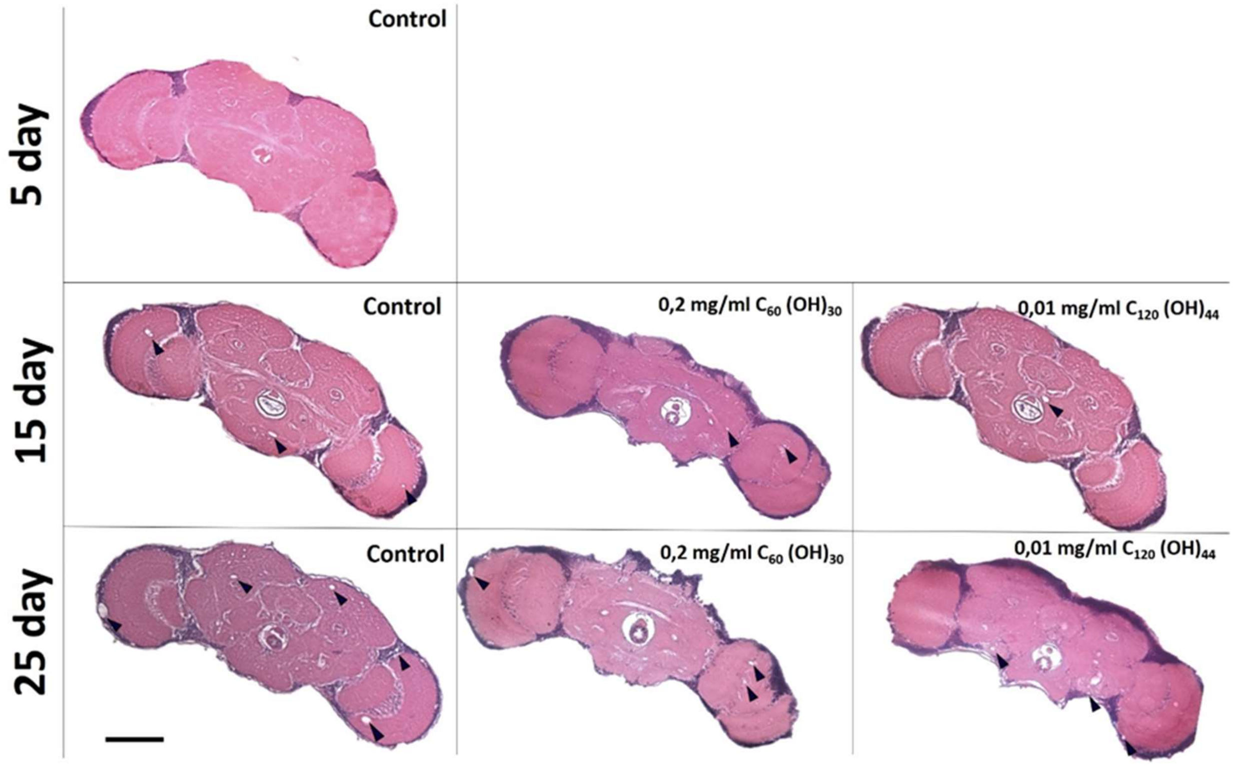

Figure S7. Paraffin brain section of the flies with HTT.128Q expression in neurons at different ages. The tip of the triangle shows a few vacuoles. Scale bar: 100 μm.
